# A sustainable food future

**DOI:** 10.1098/rsos.230702

**Published:** 2023-08-23

**Authors:** Peter Horton

**Affiliations:** School of Biosciences, University of Sheffield, Sheffield, UK

**Keywords:** food system, sustainability, environment, food security, agriculture

## Abstract

The adverse environmental impacts of food production, the ill-health resulting from excess consumption and malnutrition, and the lack of resilience to the increasing number of threats to food availability show that the global system of food provision is not fit for purpose. Here, the causative flaws in the food system are identified and a framework presented for discovering the best ways to eliminate them. This framework is based upon an integrated view of the food system and the socio-economic systems in which it functions. The framework comprises an eight-point plan to describe the structure and functioning of the food system and to discover the optimum ways to bring about the changes needed to deliver a sustainable food future. The plan includes: priorities for research needed to provide options for change; an inclusive analytical methodology that uses the results of this research and incorporates acquisition, sharing and analysis of data; the need for actions at the local and national levels; and the requirements to overcome the barriers to change through education and international cooperation. The prospects for implementation of the plan and the required changes in the outcomes of the food system are discussed.

## 1. Introduction—the defective food system

**Figure 1.**
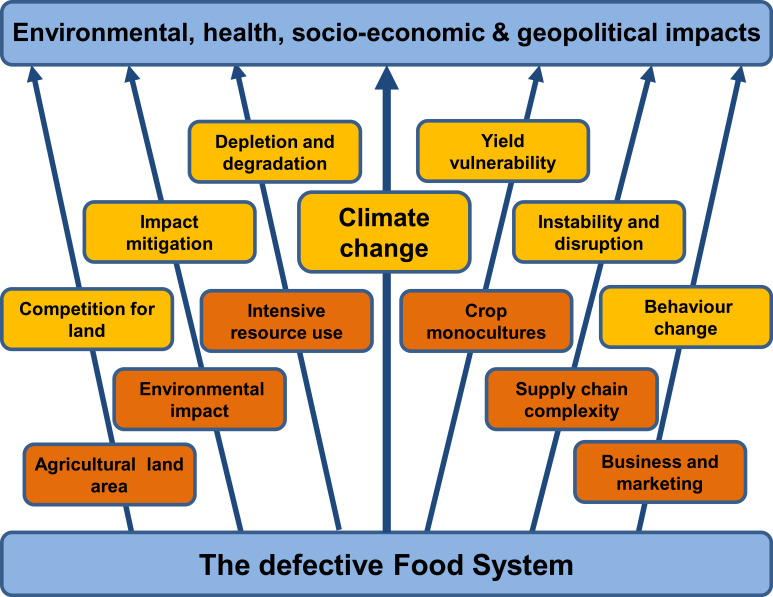
The defective food system. Several flaws (orange) give rise to multiple impacts, often exaggerated by threats arising from future trends (yellow). Climate change affects all parts of the food system, acting upon many flaws and enhancing threat levels.

The terrestrial food system since the middle of the twentieth century is one in which ever-growing demand fuels increasing levels of production, creating a system that delivers safe, nutritious and affordable food to billions of people. This was enabled by the Green Revolution and its aftermath in which increases in crop yields are achieved through development of new varieties of crops and the deployment of chemical fertilizers, pesticides and innovative farming technologies including irrigation and mechanization [1]. Similar advances in the rearing of livestock add to food production, aided by using crops as animal feed. The reduced need for agricultural workers resulting from these increases in the efficiency of food production enabled rapid urbanization and was a key factor in the increase in human prosperity. Despite these successes, there is evidence of a series of flaws which render the system unsustainable and lacking resilience, leading to several failures. These failures include not only the impacts on health from over-consumption and environmental degradation, but also inequality of provision resulting in food poverty and associated socio-economic and health impacts for a large section of the population (figure 1).

An assumption, one that has plagued other human activities, is that the Earth resources supporting the food system and the capacity of the Earth to absorb its impacts are both limitless, setting in place the first flaw: an environmentally unsustainable production system which causes greenhouse gas (GHG) emissions, pollution of land and water, depletion of groundwater, clearing of grasslands and forests and reduction in biodiversity. It is also seen as a factor in the emergence and impact of zoonotic disease such as COVID-19 [2]. Hence, the food system exceeds many planetary boundaries [3]. Moreover, it contributes significantly to the two major challenges facing humankind—climate change [4] and water security [5]. Indeed, it is argued that it will be impossible to meet these twin challenges without reform of the food system.

There are other flaws. The quest for increased yield inevitably meant that only a small number of crop species (and genotypes therein) are used, mainly rice, wheat and maize. The decreased diversity of crop plants increases the risk of disruption from pests and disease, creates genotypes highly dependent on the above inputs, and impinges on the culture, health and social economics of many areas of the world [6]—the second flaw.

A further series of flaws arise from the complexity and globalization of the global food system [7]. Vulnerability arises where supply chains converge and become ultra-sensitive to disruption [8], often due to the concentration of food production or materials needed for food production in a small number of countries and to the domination of the supply and distribution of foodstuffs by a relatively small number of large multinational companies. While complexity confers stability and resilience it could cause collapse if certain tipping points are reached [9]. This complexity has many causes but principally it derives from the activities of the food suppliers offering greater and greater consumer choice, for instance dispensing with restrictions of seasonality, demanding security of supply from producers and competition to keep prices low, all of which contribute to over-supply and waste [10]. Added to this is the composition of processed foods that contain large numbers of ingredients, which are sourced from around the globe in whatever way that maximizes profit. A further consequence of the commercial food system is the over-stimulation of demand, resulting in excessive consumption, a neglect of nutritional quality and the ill-health that results [11], and another cause of food waste. The separation of rural food production from urbanized food consumption fosters a lack of awareness of the origins of food and embeds the notion of food as a consumer item rather than a basic human need, again fuelling excess levels of consumption and waste [1]. Conversely, the financializaton of the food system leads to inequality in food availability and affordability, food poverty arising mainly in less developed countries.

Complexity and globalization also have consequences for the producers of food. Farmers may struggle to adapt to changes in costs or contracts, while the same pressures may create poor conditions for agricultural workers, which in high-income countries are often temporary migrant workers. Similarly, resource-intensive agriculture in low- and middle-income countries for export to urbanized high-income countries may force indebtedness, resource depletion and environmental impacts that are not associated with domestic consumption.

## 2. External threats to the food system

The adverse outcomes arising from these multiple flaws in the global food system can be exacerbated by a range of often uncontrollable external threats—natural disasters, extreme weather events, new trade deals and regulations, economic upheavals and political actions (figure 1) [12]. These threats can have predictable causes or may arise from unpredicted events. Others result from planned changes within the food system or in other sectors, either exactly as predicted (and desired) or as an unforeseen (undesirable) consequence. The threats have outcomes in all parts of the food system.

### 2.1. Climate change

Perhaps the most important threat to food production is climate change, including both extreme weather such as storms, flooding, heat and drought, as well as sustained changes in temperature and rainfall [13]. Climate change also exacerbates the depletion of the resources needed for crop production, especially water [14]. Incidence of pests and disease affecting both plants and animals is a continual threat to food production, one which also is predicted to be made more serious by climate change [15]. Food supply chains can also be disrupted by extreme weather events [16]. These threats from climate change affect all food-producing countries but often have a more severe impact on less resilient agriculture in low- and middle-income countries. Ironically, efforts to reduce the environmental impacts of agriculture, principally in high-income countries, by reducing the use of nitrogen-based fertilizer, restricting water use and curtailing livestock production could also reduce local food production, increasing demand for food imports, thereby transferring these impacts to other countries.

### 2.2. Land use change

A major threat arises from restrictions upon the availability and full utilization of fertile land for agriculture [17]. This has many causes, for example pressure to use land for urban development and other infrastructure. Limitation on agricultural land may also arise from environmental degradation, particularly loss of soil [18]. Land use changes arise also from measures designed to combat climate change. Thus, the push to protect biodiversity and introduce nature-based solutions to climate change will also reduce the amount of available land for cultivation [19]. Growing biofuel crops may similarly affect food crop production [20].

### 2.3. Social change

The food system is sensitive to a variety of external socio-economic factors, from political interventions, logistical problems and global catastrophes, such as the COVID-19 pandemic and the Ukraine war [21]. Such disruption can have many causes, including restricted production or supply of key food ingredients, limitations to or increased costs of energy, changes in patterns of food consumption, shortages of labour or reduced availability of materials for agrochemicals or machinery. The growing human population and the dietary transitions associated with increased economic development increase the demand for food and alter its character [22]. This is a complex issue, which is linked to changing patterns of consumption, with implications for micronutrient deficiency and other health impacts. Alterations in the food supply chain brought about by political decisions, such as the requirement for changing food sources, have been shown to bring about unforeseen changes in nutritional quality of food [23].

## 3. A framework for change

While the current trend is often to address the specific effects of flaws in the food system (often unsuccessfully), here we contend that the imperative is to correct the flaws themselves—a root and branch change in the way we produce, distribute and consume food in order to deliver the better outcomes humanity needs [1]. Such reform has been advocated repeatedly over the last 10 years (e.g. [1,22,24–29]). The global food system is a vast complex multi-layered network of interconnected components. Changes in one part have effects on other parts, often unforeseen or considered outside of the responsibility of individual operators. Thus, in thinking about how to achieve a resilient sustainable food system, it is necessary to include not only the myriad of interacting factors in the production and consumption of food, the interactions with environment and the implications for health but also the wider socio-economic, commercial, cultural and political context [30]. It is these latter factors which perhaps present the biggest obstacles to radical change in the food system.

It has been suggested that viewing the food system as a 'food ecosystem’ provides the right theoretical context [31]. Indeed, understanding the resilience of the food system relied on principles derived from the study of natural ecosystems [9,21]. Consequently, devising and evaluating any proposal for intervention to address the flaws in the food system necessitates a fully integrated approach, to consider the functions of the food ecosystem as a whole. Only a holistic ecosystem view of the food system can assess the pluses and minuses of proposals for change and the optimal trade-offs between them. So, what are the essential requirements for determining how to bring about real, effective and lasting changes that will remove the food system flaws? Below is a list of eight.

### 3.1. Commission integrated research into food systems

Research has traditionally separated the various aspects of the food system, with individual sectors setting their own priorities and objectives. It is vital that an integrated, system-wide approach is taken, so research agendas are established within the food ecosystem context, projects being co-designed by partners across disciplines. Many reforms of the food system have been discussed but most are untested and unproven within this broader view. Research is needed, principally in universities and research institutes but not in isolation: collaborations with local, national and international agencies and the private sector are essential. Funding bodies, whether governmental or charitable or commercial, need to coordinate their activities. Below are listed five key areas of research. It is imperative that they are not seen as separate but linked together in ways that provide interdisciplinary research and enable integrative solutions and policies to emerge.

#### 3.1.1. Integrated agricultural reform

Integrated thinking is needed to establish priorities for improvements in agriculture, ensuring that biologists, biotechnologists and agronomists work together—a regenerative agriculture that combines state-of-the-art plant and soil science with advanced agronomic technologies including robotics and remote sensing. Exploiting advances in plant physiology, gene editing and conventional breeding, new crop varieties can be developed that have increased yield, are more resilient and adaptable to climate change, require less fertilizer and water, and which can help preserve soils and increase carbon capture [32]. This quest includes exploiting the capabilities of a more diverse range of species, particularly N-fixing legumes [33]. Research into the structure and functions of soil is undergoing a resurgence, with crucial new areas including deciphering the roles of soil microbiota in restoration of degraded land [34] and understanding soil carbon sequestration [35]. These innovations in plant and soil science should be linked to the development of new farming practices and land management. A good example is the proposal to use ground basalt-type rocks as soil additives to increase carbon capture through enhanced rock weathering and at the same time stimulate crop growth through a multitude of fertilization effects [36]. Modifying fertilizer regimes to optimize yield and reduce environmental impact has also shown promise [37]. No-till agriculture in combination with plants that have enhanced photosynthesis, increased root storage capacity and elevated nutrient content could increase yield, carbon storage and nutrition [38]. Similar innovations are required for livestock rearing—it is generally accepted that the future food system must include a reduction in production of meat and dairy products, but research is also required on how to reduce GHG emissions from animals, repurpose animal waste for energy and fertilizer, and introduce mixed-use farming. Fully integrated agriculture has the potential to simultaneously increase food production, conserve water, reduce net GHG emissions, reduce flooding risk, increase biodiversity, preserve or enhance rural economies and provide health benefits through recreation. Whether this is through combining activities on the same land area (land sharing) or concentration of intensive agriculture in specific smaller areas (land sparing) needs to be fully assessed in all cases [17]. Agroforestry combining food production with forest management could give a similar range of co-benefits. How agriculture is practised by indigenous peoples represents a huge largely untapped source of knowledge that could also prime innovation [39].

#### 3.1.2. Food technology

Synthetic food could potentially totally transform the food system, eliminating most environmental impacts [40]. However, such a transition would have implications for farming, land use, food businesses and global supply chains, and these need to be properly assessed. How synthetic foods could contribute to sustainable food production at scale must be better understood in terms of resource use and energy consumption. The use of insects to provide both animal feed and food for human consumption is another area of promise that needs in-depth study [41]. Finally, innovations in different methods of food production such as local community-based urban agriculture, and production in controlled environments, such as vertical farms, need further exploration and development [42]. All such technologies must be assessed for scalability, economic feasibility, food safety and their contributions to human health.

#### 3.1.3. Food system structure and function

Building on recent advances [7–10,12,21], more research is needed to understand food supply chain dynamics—what are the most resilient sustainable supply chain structures, what is the optimum balance of local and global supply chains, how are economic costs incorporated into reformed supply chains and what business models best embed sustainable resilience? Thus, it is necessary first to know where in a complex food supply chain a change will bring the biggest increase in resilience or reduction in environmental impact. For example, audit of GHG emissions throughout a wheat-bread supply chain indicated the hotspots, the potential targets for remedial action [43]. Similarly, analysis of where within a supply chain food loss and waste occurs is the first step in establishing the most efficient way to eliminate it [44]. Life cycle Assessment (LCA) is central to this but must be taken much further than how it is most frequently employed [31]. Key problems to overcome are the fragmentation of the food system, its complexity and variability, and difficulty of obtaining data from all participants. Supply chains are complex and often opaque to many of the participants. Further, many parts of the food system are difficult to quantify. Finally, there is the problem of where to draw system boundaries, and how to incorporate the interactions between the food system and other sectors of society and economy. More broadly, the objective of LCA and other analytical tools such as network analysis must become the extraction of knowledge from data: to understand how the food system works; determine how it can go wrong; and discover how to protect the vulnerable parts of the supply chain [45].

#### 3.1.4. Socio-economics of the food system

The food system is central to political, cultural and economic activity, meaning that issues outside the supply chain itself must be considered. Research is needed to help meet two objectives. Firstly, we need to understand how reforms of the food system can provide not only sustainable and resilient food security but also protect culture and livelihoods. These reforms need to comply with international trade agreements and environmental regulations, but also the ambition of nations to be more self-sufficient in food. What kinds of new business models can meet these aims while ensuring profitability and stability? What interventions are needed by governments to drive these reforms? Secondly, we need to better understand the cultural, economic, environmental, political and social impacts of reduced availability of affordable food, and how to offset them. Some of these are well known and thoroughly documented, such as increased hunger, malnutrition and ill-health. Others are a consequence of the increased costs of food and include reduced business profitability, trade imbalance and suppressing growth of the wider economy. There are many social impacts—unemployment, increased inequality, civil unrest, migration and conflict between nations. As recently discussed with respect to climate change impacts, economic, political and social changes are hard to quantify and accurately estimate, adding to the potential risk [46]. Again, we need to discover how governments can implement policy to mitigate these impacts and adapt societies to effects of the reformed food system, especially to the likely increased food costs. Equally important is research to understand how to achieve the necessary shifts in food consumption. These shifts differ fundamentally between high- and low-income countries, but in all cases include the dual interlinked aims of improving the health of people and the planet. Measures to achieve these aims include improving access to food, reducing excess food consumption, reducing waste and sourcing more diverse sources of calories and protein. Reducing meat and dairy consumption is advocated as a major way to reduce the environmental impact of the food system, but this may not be relevant or applicable in areas of food poverty. How to achieve these types of significant and lasting changes in human behaviour in different societies remains a major challenge for social science.

#### 3.1.5. Agricultural land management

Agriculture uses approximately half of the Earth's habitable land [47]. New approaches to land management policy locally, nationally and globally are needed [48]. A priority is consolidating and standardizing the technologies now available for quantitative assessment of the geographical distribution of the determinants of food availability: environmental factors controlling crop yield such as temperature, rainfall, soil quality and the intensity and duration of solar radiation. Added to this we need a methodology to quantify the socio-economic factors operative in the food system such as logistics (proximity to transport links, markets etc.), availability of labour, and the cultural and political context. These factors are not fixed: genetic improvement extends the adaptation range of crop species to environmental factors; societal change alters the quantity and nature of both food demand and production; and climate change shifts environmental boundaries. It is also necessary to discover rigorous methodologies to quantify the other uses of agricultural land, for instance to assess how much GHG mitigation can be achieved without reducing food security [49]. Some of the most important questions about the food system are raised by such assessment: which land areas are best used for agriculture; should the most productive agricultural land be conserved exclusively for food production; and should less productive land that delivers low yield and/or requires excessive inputs of water and fertilizer be abandoned or diverted to other uses? Research is urgently needed to provide the rationale to answer these questions, which raise fundamental ethical and social issues that would be involved in any re-allocation of land, and the resulting effects on the livelihoods of the people affected.

### 3.2. Develop a methodology for acquiring and evaluating evidence

Outcomes of research then need to feed into an effective science–policy interface [50]. It is important to establish key principles involved in the gathering, synthesis and scrutiny of the evidence in order to establish what changes are best and discover how to develop policy to implement them. Most importantly, it is necessary to get agreement about this process from within a broad spectrum of interests, in particular the major food-producing and consuming countries, and the multinational food companies. The objective here is that this process becomes a food system standard practice and thus can drive change in all parts of the food system including the business sector. It will require input from academia, business, government agencies, international bodies, NGOs and civil society, and incorporate local, national and global influences. It is not sufficient to confine participation to ‘expert groups’—of course, such specialists are responsible for gathering and synthesizing evidence but, in addition, input is needed from ‘competent outsiders’ [51]. It is also necessary to redefine what we mean by expert to include people with hands-on local knowledge, including indigenous peoples with extensive embedded expertise about food systems in their areas [39].

**Figure 2.**
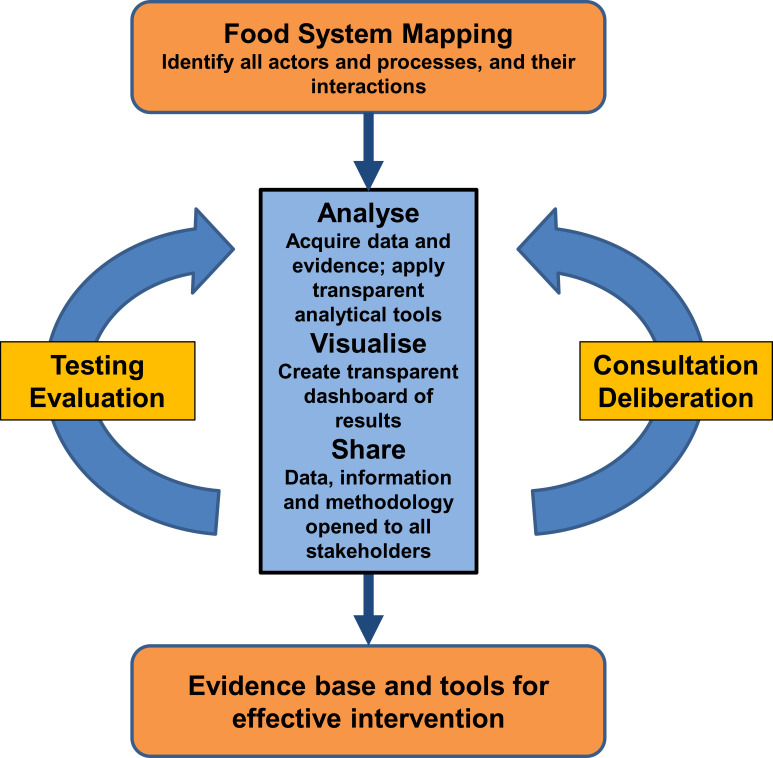
The science–policy interface for food system change. A process for acquiring and evaluating evidence as the prerequisite for policy development. See text for further details.

Figure 2 indicates how this could work. It is based on the Map, Analyse, Visualize and Share protocol [31] together with a two-pronged mechanism for scrutiny of the evidence obtained, by both independent experts and by deliberation within assemblies and groups of stakeholders and informed citizens, a process of co-development [50]. The outcome of this process is evidence in an agreed and validated form that can drive change through new policy. The process acts within and across levels—global, regional, national and local, in different food sectors and at different scales. To work effectively, information is communicated downward across levels but also fed back from below, a significant challenge (see §3.7). This process fulfils the requirement for a new science–policy interface for food systems that ensures credibility, legitimacy and diversity [52].

Many barriers must be overcome, particularly the problem of fragmentation. Fragmentation arises for many reasons. It is easier to consider a single process such as a supply chain or part of a supply chain, and it is often seen to be more economic for businesses. Organizations such as government departments or international agencies often prefer to deal with problems within narrow boundaries because it is simpler and enables retention of control and power. The need for integration is often not thought necessary, and there are frequently no mechanisms through which integration can be realized. Sharing and collaboration may contravene competition laws or confidentiality, either personal or corporate, or may be commercially sensitive. Further, this kind of cooperation may be countered by the policies of individual enterprises or governments who instead pursue activities which then exacerbate the flaws in the food system. Of particular concern is that there is often seen to be no advantage to the various participants to collaborate or to share information in the way needed.

In the following sections, the requirements that need to be met to establish the MAVS protocol for operation of the food science–policy interface are listed.

### 3.3. Establish terminology and metrics

It is necessary to unambiguously define and distinguish resilience and sustainability. A resilient food system is not always sustainable—for example, a plan to increase domestic food production through extending intensive industrial agriculture may increase resilience but may not be sustainable. Equally, it is important to define sustainability in its broadest sense and not just in terms of environmental impact [53]—for example, transition to organic farming practices can in some cases reduce environmental impact but would not meet other aspects of sustainability, such as reducing hunger.

The next step is to devise metrics of sustainability and resilience. Discovering a successful intervention in the food system first requires reliable, rigorous and meaningful metrics of the multidimensional outputs of food provision: how exactly do we measure when a process or an activity is sustainable; how do we reliably offset sustainable and unsustainable activities (including trade-offs and compromises); how do we measure the scale up from individual actions to national and global dimensions; and how do we quantify the various external criteria defining a successful sustainable food system, preservation of biodiversity, human health and well-being, and humanity's harmony with nature? The difficulty of answering these questions is exemplified in a recent study of the plan for expansion of bioenergy use in the EU, and its impact on regional and international land use for agriculture, biodiversity protection and carbon storage goals [54]. The difficulty in agreeing metrics of biodiversity is hampering international efforts in that domain [55], and reliably quantifying carbon offsets is a major focus of climate change negotiations [56]. These problems are linked to the issues surrounding the inadequacy of GDP to define economic success and the argument that economic growth should include metrics that describe impacts on people, ecosystems and climate [57].

There are already initiatives striving to devise useful metrics of the environmental and health impacts of foods. Indices combining health and environment are being used to construct dietary choices [58]. Large number of foods have been classified according to nutrition and environmental impact as a prelude to incorporating into food labelling [59]. As discussed above (see §3.1.3), these kinds of metrics can be incorporated into new developments in life cycle assessments, as the starting point for constructing sustainable and resilient food chains.

### 3.4. Share data and intellectual property

Food system reform requires the collection, analysis, synthesis and validation of large amounts of evidence and data obtained from, and shared between, a wide variety of sources within the food system. The revolution in the production and analysis of data provides an unprecedented opportunity to advance the development of integrated food systems approaches. It is now feasible to use data to describe in fine granular detail the mechanics of food systems, the human behaviours that drive them, and their socio-economic consequences. Within the global food system there are a huge number of different supply chains, agricultural practices and food delivery processes—within these are key determinants of what is most efficient, sustainable and resilient. Through data science including AI it will be possible to develop of holistic, system-wide maps to describe all these features of the structure and function of food systems.

There are many barriers to break through to establish the trust necessary for this level of data use [60], particularly with respect to AI [61]. These include how to obtain and record the required data, monitor accuracy, remove bias, ensure transparency, prevent manipulation and maintain ethical practice. How to encourage (and/or enforce and regulate) data sharing and exchange remains a key obstacle [62]. Another difficulty concerns the lack of standardization of methodologies for data collection and analysis from multiple, often diverse, sources. This last requirement is particularly challenging as found in the public health sector; e.g. determining the number of deaths from COVID-19 globally has proved impossible because of different systems of recording and interpreting data [63]. Finally, even if these barriers can be overcome, there is the problem on how best to communicate vast amounts of often complicated data [64].

There is already a lot of activity around this issue in other sectors, within the EU and USA [65]. The health sector is leading the way, catalysed by the increased involvement of digital technologies in diagnosis and online treatments, and the use of health data to understand health promotion and disease prevention at the population level [66]. WHO is promoting the sharing of new treatments for disease, aided by pharmaceutical companies and non-governmental organizations (NGOs). Many of these innovations and strategies could be applied to the food system. The FAO aligned with CGIARs must continue to mobilize the dispersal of new agricultural and other food-related biotechnologies, working with international aid agencies. Private sector involvement is crucial—this sector makes major advances in food technologies that principally benefit the high-income countries which house the innovators [67], but ways must be found to bring benefits to low- and middle-income countries where threats to food availability are greatest.

### 3.5. Use digital technology

There are now much-improved opportunities to use AI and digital modelling techniques to predict the behaviour of complicated systems. In biological and biomedical research, profound breakthroughs are enabled, from predicting the structure of proteins from their primary amino acid sequences to understanding how multiple genes control cell function and disease states. In climate science, more accurate predictions of the effects of GHG emissions on global warming and changing weather patterns are enabled. Increasingly refined models have been used in formulating policies to deal with the spread of COVID-19. These techniques enable the use of data to model food systems and facilitate prediction of their resilience and the impact of future trends.

**Figure 3.**
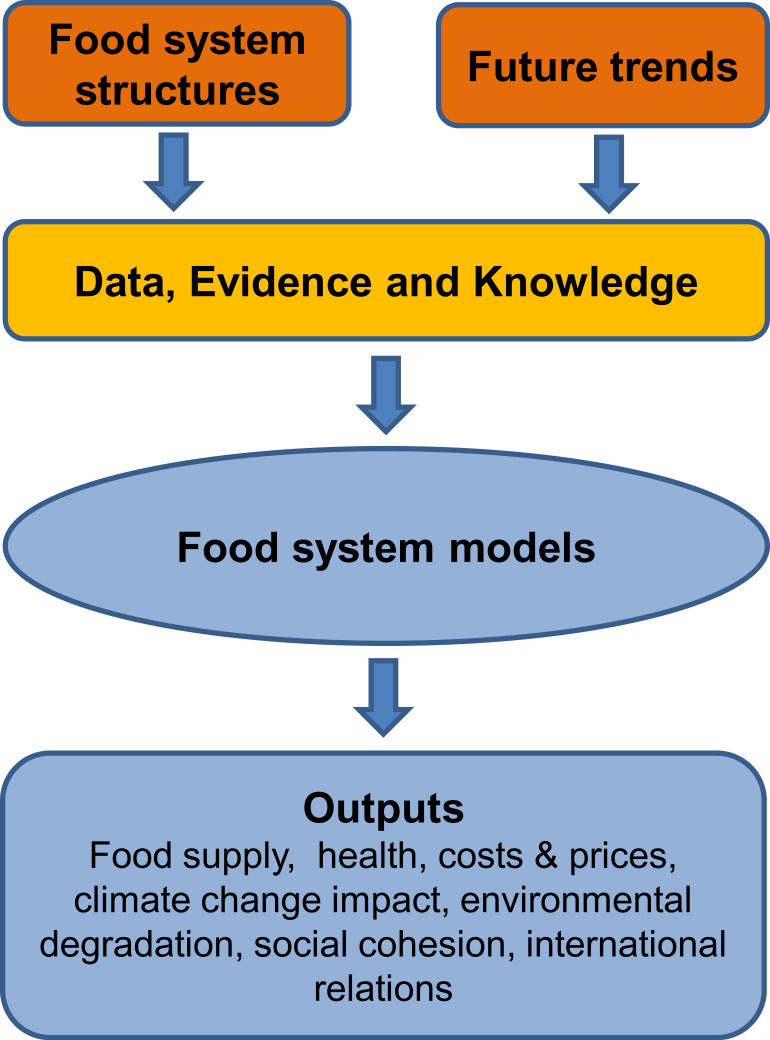
Predicting food system outcomes, resilience and sustainability. The data and evidence made available as in §3.4 from mapping of food systems (§3.2) can be used to construct digital models of food system functioning that enable prediction of the response to external threats and the outcomes resulting from proposed changes to the food system.

The objective would be to create digital twins of the food system [68]. These will enable the effects of threats and the potential of interventions to bring about change and assess their impact in future scenarios and incidents (figure 3). Complicated supply chains and the numerous interactions between them and with other economic sectors can be incorporated. Waste and loss, GHG emissions, resource-use efficiency, costs and prices, impacts of health and so on can be the outputs. Individual food products can be assessed in fine granular detail. Food availability at the individual, local, national and global scales can be mapped and evaluated, the consequences of disruptions and interventions. Models must be of different types to suit the user—advanced models to inform international bodies, to aid policymakers in government, or to help develop business strategies together with practical user-friendly tools to help consumers, farmers and small businesses make informed choices.

### 3.6. Improve and exploit education and communication

The transformation of the food system depends on people—consumers, employees in food businesses, farmers, members/advisors of NGOs, international organizations and governments. Education in all aspects of the sustainable society, and specifically related to the food system, underpins everything discussed in this article. It is important to find ways to promote an understanding of evidence and facts, of how the scientific process works so people not directly involved in the food system can make informed choices and influence both government and business, becoming what have been described as competent outsiders [51]. Communication, through mainstream media, social media, art, music and literature is a crucial part of this process of driving change through education [69].

These requirements vary across different countries. In some, improving gender equality in education is a priority: there is evidence that equal access by women to education, training, resources and finance together with equal rights and status can bring about positive changes in the food system, given the dominant role they play [70]. In others, schools, colleges and especially universities must play their part, for example, offering courses in the innovative analysis of food supply chains as a part of programmes of teaching and learning in all aspects of sustainability [71]. The fact that assessment of the quality of universities worldwide now includes their contribution to delivering sustainable development goals indicates such changes are already having impact.

The food system has undergone many changes in the right direction: increased availability of meat-free products, improved animal welfare, awareness of food waste and unnecessary packaging and so on. The extent to which these changes result from better-informed consumers making altered food choices, or from public or political pressure on food companies (and their shareholders), or from these companies recognizing the need for change and identifying new ways to increase profit is currently unclear, but in all cases could be the outcome of better ‘education’.

### 3.7. Promote and coordinate local, national and international action

What needs to be avoided is creation of new vast centralized top-down bureaucratic bodies attempting to impose change universally. Collective action agreed across all countries is needed because all nations share responsibility for global food security and the functioning of global supply chains. However, food security is not the same type of issue as climate change—national action cannot alter the climate of that nation specifically, unlike interventions within the food system, where in theory effective changes can be initiated at the local or national levels. Indeed, it has been argued that food system reforms are best approached initially from the national perspective [72]. Thus, the emphasis must be on locally driven actions (small businesses, farmers, communities) but most importantly informed by validated evidence. Local actions can then be networked, contributing to and driving change at the national level. A good example of how this could work nationally is found in the UK's Food Industry Resilience Forum, set up in response to the disruption of food supply chains during the COVID-19 pandemic. The various bodies which advise and direct policy on the diverse aspects of the food system must also be better coordinated and if necessary consolidated—in health, environment, business, trade and agriculture—to give coherence and allow integrated thinking and actions. There is evidence that only by coordination across the different government departments can effective national action result. Thus, in China, the move to switch to different crops only led to the desired enhancement of environmental sustainability and improved farmer incomes when all relevant ministries worked together [73]. However, there are restrictions on the scope of change at the national level, because of the global supply chains, global commodity markets and the operations of multinational food companies. Thus, it is important that collaboration occurs between nations in the same ways as within nations, each learning from the other and coordinating their actions. There are other advantages of this: the problem of dealing with the adverse environmental impacts of food produced in one country and consumed in another can be remedied through collective agreements and actions. What international administrative structure is needed to enable such collaboration is one of the most urgent as yet unanswered questions. Encouragingly, agriculture and food are becoming increasingly prevalent in UN deliberations about climate change, biodiversity and water use, but such activities need to be better coordinated and integrated. The World Bank's Food Security updates and its recent Global Forum for Food and Agriculture event entitled Food Systems Transformation: A Worldwide Response to Multiple Crises provides a model upon which concerted international action can be built.

### 3.8. Develop mechanisms for compliance

National governments must be the principal agents for compliance, setting objectives and priorities for action. There are many possible mechanisms, ranging from voluntary to enforced and comprising persuasion, incentives, rules and penalties. Much can be learned from exploring the problems involved in making the changes needed to combat climate change. It has been argued that new financial regulations are needed to enforce disclosure of climate risks [74]. However, the effects of mandatory compliance can have unforeseen consequences that vary from region to region and sector to sector [75]. Different countries with different legal and political structure have different capabilities to develop and implement policy [76]. Sustainability and resilience must somehow become incorporated into the ethics of both the food business practice and consumer behaviour. Trade agreements must similarly include these criteria, with WTO monitoring compliance. A key part of compliance is the availability of reliable data supporting accepted metrics of sustainability and other impacts. All too often various stakeholders are found exaggerating claims of sustainability, ‘greenwashing’ being common in many sectors, including food. The processing of data through AI and the increasing availability of technologies to monitor directly environmental impact from remote sensing will increasingly make such diversion tactics more difficult.

## 4. Conclusion—prospects for implementation

**Figure 4.**
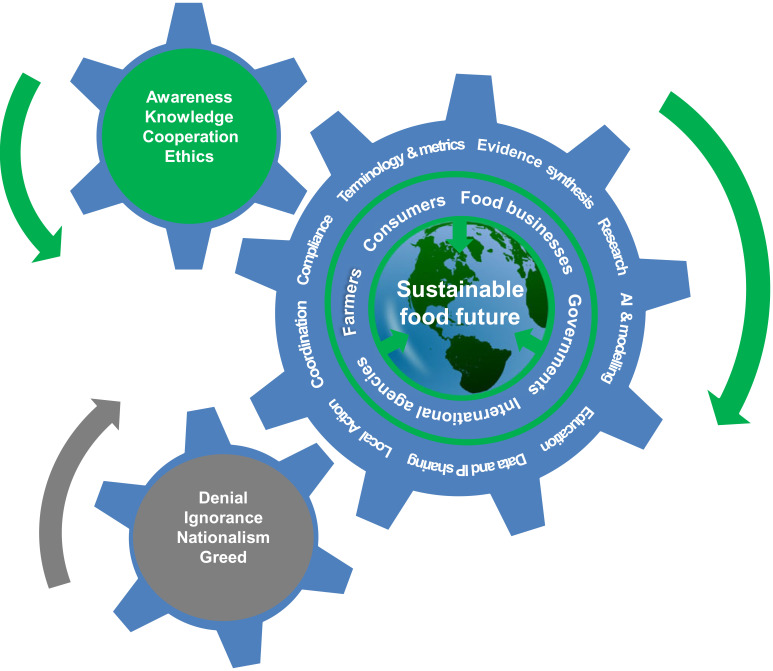
Framework for delivering a sustainable food future. The processes and actors in the eight-point action framework (blue) are driven by positive societal forces (green) but opposed by negative forces (brown). See text for further details.

It is broadly agreed that the food system needs transformation if it is to deliver global food security while removing its contribution to GHG emissions and helping humankind stay within planetary boundaries. As noted above there is already evidence of positive changes within the food system. But these are still limited in scale and scope. Various negative forces delay and subvert fundamental change within the food system (figure 4)—nationalism giving rise to protectionist actions, and self-interest which drives governments, businesses and individuals to pursue only their own short-term goals. The motivation may stem from denial of evidence or ignorance of facts but more often is based purely on maintaining power and most of all, increasing wealth. The situation is very similar to the role the fossil fuel industry and fossil fuel-producing countries have played in undermining and delaying international efforts to reduce climate change. The intensive financialization of the food system, and the associated lobbying and pervasive influence are therefore perhaps the major obstacles to change [77]. The framework presented here must work against these negative forces. Each of the areas of action within framework are driven by a collective desire to deliver a sustainable and resilient food system, all derived from an awareness of the severity of the problem, from knowledge of the food system, but also from a desire for cooperation and from standards of morality, ethics, justice and social conscience.

There is evidence across countries and societies of increasing awareness of all aspects of sustainability and of the need for change [69], and as discussed above, the food system is increasingly seen as a key area for reform. As the world moves closer to catastrophe from climate breakdown, as more and more planetary boundaries are crossed, as the commercial viability of the food system is put at risk, the need for reform will become irresistible. This framework, informed by new research and innovation, built on inclusion, consensus and cooperation provides a plan for discovering and defining the best ways to deliver this transformation, driving change to the structure and functioning of the food system, and delivering a sustainable food future.

## Data Availability

This article has no additional data.
